# Meta-analysis of postoperative incision infection risk factors in colorectal cancer surgery

**DOI:** 10.3389/fsurg.2024.1415357

**Published:** 2024-08-13

**Authors:** Li Jia, Huacai Zhao, Jia Liu

**Affiliations:** ^1^ Department of Infection Control, People's Hospital of Dayi County, Chengdu, Sichuan Province, China; ^2^ Department of Urology, People's Hospital of Dayi County, Chengdu, Sichuan Province, China; ^3^ Department of Infection Control, Chengdu Fifth People’s Hospital, Chengdu, Sichuan Province, China

**Keywords:** colorectal cancer, postoperative incision infection, risk factors, meta-analysis, laparoscopic surgery

## Abstract

**Objective:**

To evaluate the risk factors for postoperative incision infection in colorectal cancer, this meta-analysis aimed to identify key variables impacting infection incidence following colorectal cancer surgery.

**Methods:**

Utilizing a meta-analytical approach, studies published from January 2015 to December 2022 were systematically collected and analyzed through the assessment of factors like body mass index, diabetes, albumin levels, malnutrition, and surgical duration.

**Results:**

The meta-analysis of eleven high-quality studies revealed that elevated BMI, diabetes, low albumin levels, malnutrition, and extended surgical duration were associated with increased infection risk, while laparoscopic procedures showed potential for risk reduction.

**Conclusions:**

This study underscores the significance of preoperative risk assessment and management in mitigating postoperative incision infections in colorectal cancer patients. The findings present actionable insights for clinicians to enhance patient prognoses and overall quality of life

## Introduction

Colorectal cancer ranks among the malignancies with the highest incidence and mortality rates globally, particularly in developed countries (1, 2). As a significant type of gastrointestinal malignancy, colorectal cancer spreads through lymphatic and blood circulation, imposing significant physical and psychological burdens on patients (3–7). According to recent cancer statistics, colorectal cancer ranks third in incidence and fifth in mortality among all cancers in China, with an annual report of 376,000 new cases and 191,000 deaths (8–10). These figures highlight the substantial impact of colorectal cancer on individuals and society and underscore the importance of timely and effective diagnosis and treatment (11–13).

Clinically, surgical resection remains the mainstream treatment for colorectal cancer, with curative surgery being the standard treatment strategy (14–16). Although such surgery can control disease progression to some extent, numerous studies have identified postoperative incision infection as a standard and severe complication, which, in severe cases, may lead to sepsis, systemic infection, and death (17–19). Advances in medical technology have made laparoscopic surgery the preferred technique for treating colorectal cancer due to its minimally invasive nature, fewer complications, and faster postoperative recovery (20–24). However, the risk of postoperative incision infection persists, affecting patient recovery speed, increasing treatment costs, and exacerbating the burden on healthcare resources (25–27).

Patients undergoing surgery for colorectal cancer face a heightened risk of postoperative incision infection due to the necessity for prolonged fasting and bowel preparation preoperatively and the potential for contamination of the surgical area by intestinal contents during surgery (28–30). Furthermore, the invasion of pathogens directly causes these infections (31). In China, the incidence of surgical site infections reaches as high as 1.01% (32–36), with rates in specific regions and populations potentially soaring to 20%, especially in resource-constrained developing countries (14, 37–39).

Although extensive research has been conducted on the risk factors for postoperative incision infection in colorectal cancer, findings remain varied without a unified conclusion (40–42). Factors such as age, medical history, and surgical duration are considered potential influencers of infection risk, yet their relative importance and interactions still need to be fully clarified (43–45). Despite attempts to explore preventative measures, the effectiveness of prevention and treatment strategies requires further research due to issues like insufficient sample sizes and study design biases (46–48).

This study aims to provide a more precise risk assessment and effective prevention strategies for clinical application, thereby improving patient recovery quality and reducing associated medical costs through systematic analysis and meta-analysis of various risk factors for postoperative incision infection in colorectal cancer. Beyond focusing on the direct risk factors, this research also examines how optimizing preoperative preparation, improving surgical techniques, and enhancing postoperative management can effectively reduce the incidence of incision infection. The findings guide clinicians in devising individualized treatment plans, enhancing surgical safety, and improving patient quality of life. Additionally, reducing postoperative incision infections can significantly lower medical costs, alleviate the economic burden on patients and their families, and improve healthcare service quality and patient satisfaction. In summary, this study aims to create a safer and more effective surgical treatment environment for colorectal cancer patients through in-depth analysis and comprehensive evaluation.

## Materials and methods

### Literature source and retrieval

This study searched for risk factors associated with postoperative incision infection in colorectal cancer patients across Chinese and English databases, including VIP, Wanfang, CNKI, PubMed, EMBASE, and DSR. The search strategy involved selecting relevant literature based on inclusion and exclusion criteria. The Chinese search formula included combinations of terms for “colorectal cancer”, “rectal cancer”, or “colon cancer”, with “surgical site infection”, “incision infection”, and “risk factors”, or “influencing factors” or “related factors”. The English search strategy used “colorectal neoplasms” and “surgical wound infection” combined with “risk factors”, “influence factors”, or “dangerous factors”. Searches were tailored by combining phrases freely and, when necessary, seeking related literature—the search period spanned from January 2015 to December 2022.

### Inclusion and exclusion criteria for literature

Inclusion criteria for the literature were: (1) Studies addressing risk factors or influencing factors for postoperative incision infection in patients with colorectal cancer. (2) Clinical studies in the form of case-control or cohort studies. (3) Studies involving at least 30 patients. (4) Studies involving patients aged between 18 and 80.

Exclusion criteria for the literature were: (1) Publications without clinical trials, such as reviews and case analyses. (2) Duplicated publications. (3) Clinical trial articles or documents with incomplete data. (4) Unpublished documents. (5) Studies with too small sample sizes in clinical trials. (6) Studies involving patients who were too young or too old.

### Literature screening and data extraction

Preliminary Screening: A primary search was conducted using combinations of keywords on major literature platforms. Eligible publications were collected and organized using Excel for categorization and sorting, removing duplicates. Initial reviews of collected titles and abstracts were performed to eliminate documents with significant differences. A thorough reading of selected literature was conducted according to acceptance and organization standards to exclude documents that did not meet research criteria, documenting the number of publications and reasons for exclusion.

Secondary Screening: Conducted independently by two researchers based on the literature's inclusion and exclusion criteria to further select and extract collected documents, documenting the number and reasons for excluded literature.

Tertiary Screening: In cases of disagreement on inclusion between the researchers above, another researcher independently reviewed and resolved discrepancies in literature selection.

### Literature quality assessment

This study conducted a literature quality assessment based on the Cochrane risk of bias tool to ensure the reliability of the research findings. Potential biases in each study, such as random sequence generation, allocation concealment, blinding, and outcome assessment, were classified into low, high, or unclear risk categories. Studies were categorized as having a high risk of selection bias if there were significant deficiencies in randomization or allocation concealment, as an unclear risk if there was insufficient information to assess the risk of bias, and as low risk if randomization and allocation concealment were appropriately conducted and blinding was adequately implemented.

Furthermore, two researchers assessed the quality of case-control or cohort studies using the Newcastle-Ottawa scale (NOS), which includes four items (4 points) for the selection of study participants, one item (2 points) for the comparability of groups, and three items (3 points) for the outcome measurement, with a total score above 9 considered high quality. The quality of cross-sectional studies was evaluated using the assessment criteria recommended by the Agency for Healthcare Research and Quality (AHRQ), comprising eleven standards such as data sources, inclusion criteria, observation period, continuity of subjects, subjective factors of assessors, and quality control. Studies scoring between 0 and 3 were considered low quality (Grade C), and those scoring between 4 and 7 were considered medium quality (Grade B).

### Statistical analysis

Meta-analysis was performed using R software, selecting r values and their 95% confidence intervals (CI) as the effect size indicators. The chi-squared (X^2^) test was used to process control trial data from all selected literature, and heterogeneity of the collected experimental data was evaluated using the *I*^2^ statistic. If *P* < 0.01 and *I*^2^ > 50%, it indicates no difference in the data across the selected literature, allowing for a fixed-effect model to combine and analyze control trial data. If *P* > 0.01 and *I*^2^ < 50%, an investigation into the sources of data heterogeneity is required, followed by relevant subgroup interventions. If the value remains large, data correlation analysis is conducted using a random-effects model, with odds ratios (OR) used for effect statistics and 95% CI for interval estimation, excluding clinical studies cited no fewer than five times in the literature.

Additionally, sensitivity analysis was employed to assess the stability of the research outcomes, with funnel plots drawn to evaluate the potential for publication bias. The significance level for all statistical tests was set at *P* > 0.05.

## Results

### Overview of literature retrieval results based on the systematic screening process

In systematic reviews or meta-analyses, the retrieval and screening of literature are fundamental steps to ensure the quality and comprehensiveness of the research. This study employed a multi-database search strategy and stringent inclusion and exclusion criteria to conduct a comprehensive search and screening of relevant literature.

**Figure 1:**
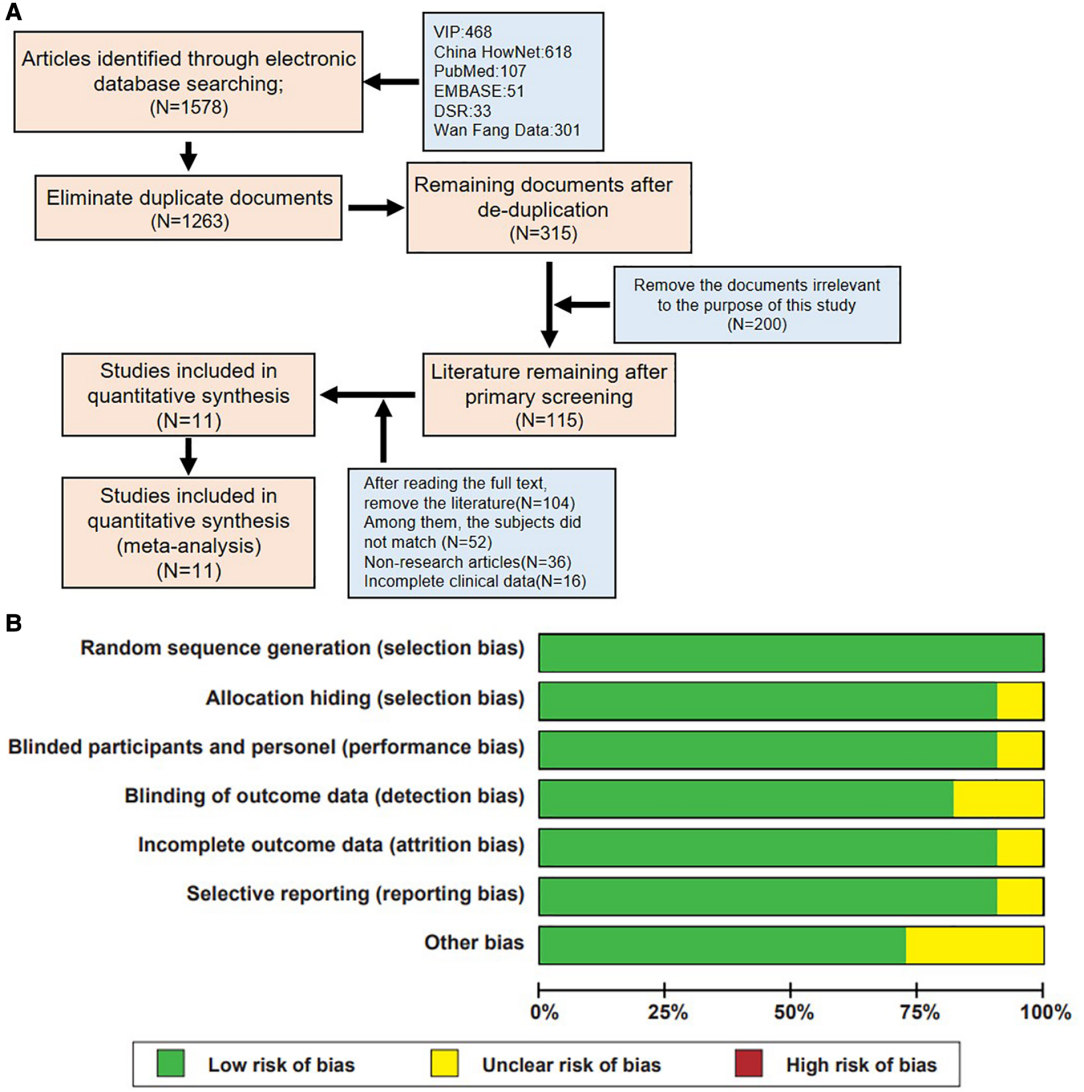
Literature screening process and quality assessment results of included studies. **(A)** Flowchart of literature inclusion. **(B)** Summary of bias risk assessment for included studies.

Initially, the search yielded 1,578 articles, including 468 from VIP, 301 from Wanfang, 618 from CNKI, 107 from PubMed, 51 from EMBASE, and 33 from DSR databases. After removing 1,263 duplicates, 315 articles remained for preliminary screening. Through careful reading of titles and abstracts, and based on the objectives and predefined conditions of the study, this number was further reduced to 115 articles. After a detailed full-text review, 11 articles met the study's inclusion and exclusion criteria (Figure 1A).

Through a rigorous literature search and screening process, this study successfully identified 11 high-quality studies from a large pool of related literature, providing a solid foundation for subsequent analysis and review. Furthermore, the quality of the included literature was evaluated based on the Cochrane risk of bias tool standards (Figure 1B), ensuring a fair and comprehensive quality review of the included studies.

### Summary of high-quality literature based on NOS scores

In various fields of scientific research, assessing the quality of literature and interpreting data are crucial. This study conducted an in-depth literature analysis within a specific domain, employing the NOS for literature quality assessment. All included studies scored ≥7 on the NOS, indicating they are of high quality, as detailed in Table 1 (8, 33, 37, 49).

**Table 1:** Characteristics and quality evaluation of included documents.

Author	Region	Research type	Case group/Exposure group (example)	Control group/Unexposed group (example)	Risk factor	Nos score
Ni et al.	China	Case control	24	38	1.2	7
Deng et al.	China	Case control	13	187	1.2.5.8	8
Wang et al.	China	Case control	60	837	1.2.5.6.7.8	7
Zhao et al.	China	Case control	15	185	2.5.6	8
Atsushi et al.	Japan	Case control	95	3,075	1.3	8
Liu et al.	China	Case control	61	660	1.2	7
Ma et al.	China	Case control	17	265	1.2.7	7
Wu et al.	China	Case control	14	134	1.4.5.8	7
Li et al.	China	Case control	12	114	2.4.5.8	8
Liang et al.	China	Case control	30	173	2.8	7
Zhu et al.	China	Case control	38	158	1.2.3.5.8	7

1: BMI ≥25 kg/m^2^; 2: diabetes; 3: placing subcutaneous drainage; 4: Preoperative low albumin; 5: surgical methods; 6: malnutrition; 7: age; 8: The duration of operation is <3 h.

### Analysis of significant risk factors for postoperative incision infection in colorectal cancer

In meta-analysis research, testing for heterogeneity among risk factors is critical in evaluating differences across studies. This process aids in identifying the most suitable effect model to ensure the accuracy and reliability of the analysis results. Our study comprehensively examined six potential risk factors, conducting a detailed assessment of their heterogeneity (Table 2).

**Table 2:** Overview of heterogeneity tests and effect model selection for various risk factors.

Risk factor	Number of documents	Heterogeneity test		Effect model
		*I*^2^ (%)	*P*	
BMI ≥24 kg/m^2^	6	97	>0.01	Random effect model
Diabetes	11	94	>0.01	Random effect model
Preoperative low albumin	4	93	>0.01	Random effect model
Operation mode	7	91	>0.01	Random effect model
Malnutrition	2	0	0.94	Fixed effect model
Operation duration <3 h	4	98	>0.01	Random effect model

*I*^2^, I-squared statistic for heterogeneity; *P*, *P*-value for heterogeneity; the *I*^2^ statistic describes the percentage of total variation across studies that is due to heterogeneity rather than chance. A higher *I*^2^ value indicates greater heterogeneity. The *P*-value tests the null hypothesis that the studies are homogeneous. The effect model column indicates the statistical model applied for the meta-analysis based on the heterogeneity test results: a Random effect model is used when significant heterogeneity is detected, and a Fixed effect model is employed when heterogeneity is low or not statistically significant.

**Figure 2:**
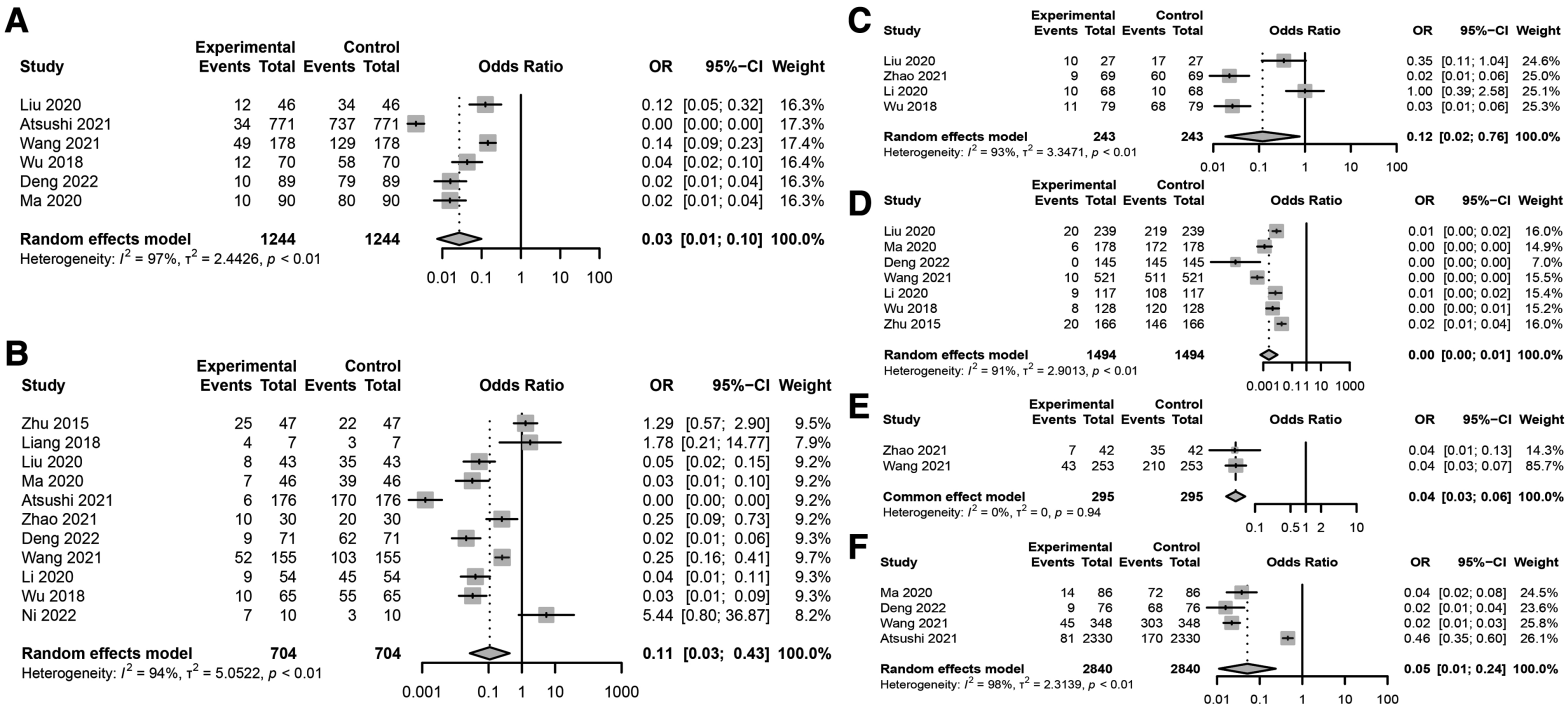
Key risk factors for postoperative incision infection in colorectal cancer. **(A)** Association between BMI ≥24 kg/m^2^ and postoperative incision infection in colorectal cancer. **(B)** Forest plot analyzing the risk of postoperative incision infection in colorectal cancer associated with diabetes. **(C)** Forest plot analyzing the impact of preoperative low albumin levels on the risk of postoperative incision infection in colorectal cancer. **(D)** Association between surgical methods and the risk of postoperative incision infection in colorectal cancer. **(E)** Association between preoperative malnutrition and the risk of postoperative incision infection in colorectal cancer. **(F)** Association between surgical duration exceeding 3 h and the risk of postoperative incision infection in colorectal cancer.

The analysis identified high body mass index (BMI), diabetes, preoperative low albumin levels, preoperative malnutrition, and surgical duration exceeding 3 h as significant risk factors for postoperative incision infection in colorectal cancer. Conversely, laparoscopic surgery emerged as a factor associated with a reduced risk of infection (Figure 2). Understanding these factors is crucial for the prevention and management of postoperative incision infection in colorectal cancer.

Specific meta-analysis findings include: a BMI ≥24 kg/m^2^ significantly increases the risk of postoperative incision infection (Figure 2A), with a combined OR of 0.03 and a 95% CI of [0.01; 0.10], indicating a significant association. However, this result showed high heterogeneity (*I*^2^ = 97%), suggesting substantial differences between included studies. Diabetes was also a significant risk factor (Figure 2B), despite high heterogeneity (*I*^2^ = 94%), with a combined OR of 0.11 and a 95% CI of [0.03; 0.43], indicating an increased risk of infection post-surgery for patients with diabetes. Preoperative low albumin levels were significantly associated with postoperative incision infection (Figure 2C), with a combined OR of 0.12 and a 95% CI of [0.02; 0.76], despite high study heterogeneity (*I*^2^ = 93%). Laparoscopic surgery appeared to be associated with a lower risk of infection (Figure 2D), with a combined OR of 0.00 [95% CI: 0.00; 0.01], even though its heterogeneity was high (*I*^2^ = 91%). Preoperative malnutrition was significantly linked to an increased risk of incision infection (Figure 2E), with a combined OR of 0.04 and a 95% CI of [0.03; 0.06], and heterogeneity testing (*I*^2^ = 0%) indicated no significant differences between studies. Surgical duration exceeding 3 h was significantly associated with an increased risk of postoperative incision infection in colorectal cancer (Figure 2F), with a combined OR of 0.05 and a 95% CI of [0.01; 0.24], and heterogeneity testing showed very high differences between studies (*I*^2^ = 98%).

Through meta-analysis, this study revealed associations between postoperative incision infection in colorectal cancer and multiple significant risk factors. Factors such as BMI ≥24 kg/m^2^, diabetes, preoperative low albumin levels, preoperative malnutrition, and surgical duration exceeding 3 h all demonstrated a significant increase in risk despite high heterogeneity. Meanwhile, the protective role of laparoscopic surgery warrants attention, though its heterogeneity calls for further investigation. These findings emphasize the importance of comprehensive patient assessment and management in clinical practice, particularly identifying and intervening in these risk factors preoperatively to reduce the risk of post-surgery infection.

### Sensitivity analysis of risk factors for postoperative incision infection in colorectal cancer shows high stability

**Figure 3:**
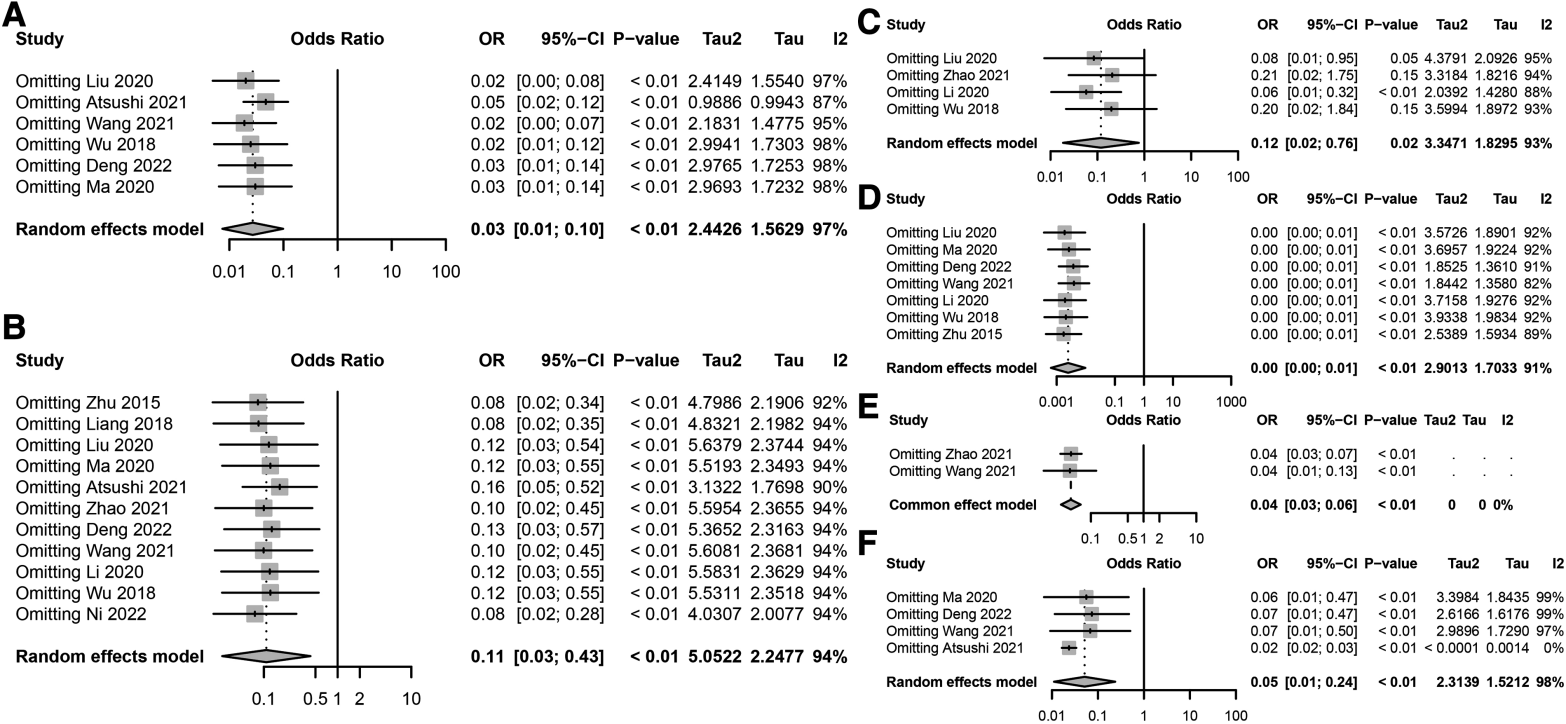
Sensitivity analysis of risk factors for postoperative incision infection in colorectal cancer. **(A)** Sensitivity analysis results for the association between BMI ≥24 kg/m^2^ and the risk of postoperative incision infection in colorectal cancer. **(B)** Sensitivity analysis results for the association between diabetes and the risk of postoperative incision infection in colorectal cancer. **(C)** Sensitivity analysis results for the association between preoperative low albumin levels and the risk of postoperative incision infection in colorectal cancer. **(D)** Sensitivity analysis results for the association between surgical methods and the risk of postoperative incision infection in colorectal cancer. **(E)** Sensitivity analysis results for the association between preoperative nutritional status and the risk of postoperative incision infection in colorectal cancer. **(F)** Sensitivity analysis results for the association between surgical duration exceeding 3 h and the risk of postoperative incision infection in colorectal cancer.

A sensitivity analysis of multiple risk factors for postoperative incision infections in colorectal cancer patients revealed significant impacts on the risk of incision infections by BMI ≥24 kg/m^2^, diabetes, low albumin levels, laparoscopic surgery, and preoperative malnutrition (Figure 3). The sensitivity analysis results of BMI ≥24 kg/m^2^ on the risk of postoperative incision infections remained significantly correlated even after excluding any individual study, with no substantial impact on the overall conclusion from the changes in the combined effect sizes (OR = 0.03, 95% CI: 0.01–0.10), indicating the stability of BMI ≥24 kg/m^2^ as a risk factor (Figure 3A). The sensitivity analysis of diabetes on the risk of postoperative incision infections also exhibited significant association even after excluding any single study, with the combined effect size (OR = 0.11, 95% CI: 0.03–0.43) confirming the robustness of diabetes as a risk factor (Figure 3B). Analysis of the sensitivity of preoperative low albumin levels on the risk of postoperative incision infections indicated a significant increase in risk, with high consistency across the study results reflected by the combined effect size (OR = 0.12, 95% CI: 0.02–0.76) (Figure 3C). The sensitivity analysis of laparoscopic surgery on the risk of postoperative incision infections revealed a significant risk reduction with this surgical approach, as evidenced by the unchanged combined effect size (OR = 0.00, 95% CI: 0.00–0.01) even after exclusion of any individual study, demonstrating the robustness of the conclusion (Figure 3D). Sensitivity analysis of preoperative malnutrition and operations exceeding 3 h on the risk of postoperative incision infections confirmed their significant increase in risk, with no substantial impact on the overall conclusion from the changes in the combined effect size and 95% confidence intervals after excluding any individual study, verifying their consistency and stability as important risk factors (Figures 3E,F). Through these sensitivity analyses, we affirmed the significant impact of BMI ≥24 kg/m^2^, diabetes, preoperative low albumin levels, preoperative malnutrition, operations exceeding 3 h, and laparoscopic surgery on the risk of postoperative incision infections in colorectal cancer patients. These findings underscore the importance of comprehensively assessing and managing these risk factors in clinical practice to ensure that no single study decisively influences the overall conclusion, even in the presence of high heterogeneity, where the changes in combined effect size and 95% confidence intervals remain insufficient to significantly affect the overall conclusion.

### Assessment of publication bias strengthens the credibility of research on risk factors for postoperative infection in colorectal cancer

**Figure 4:**
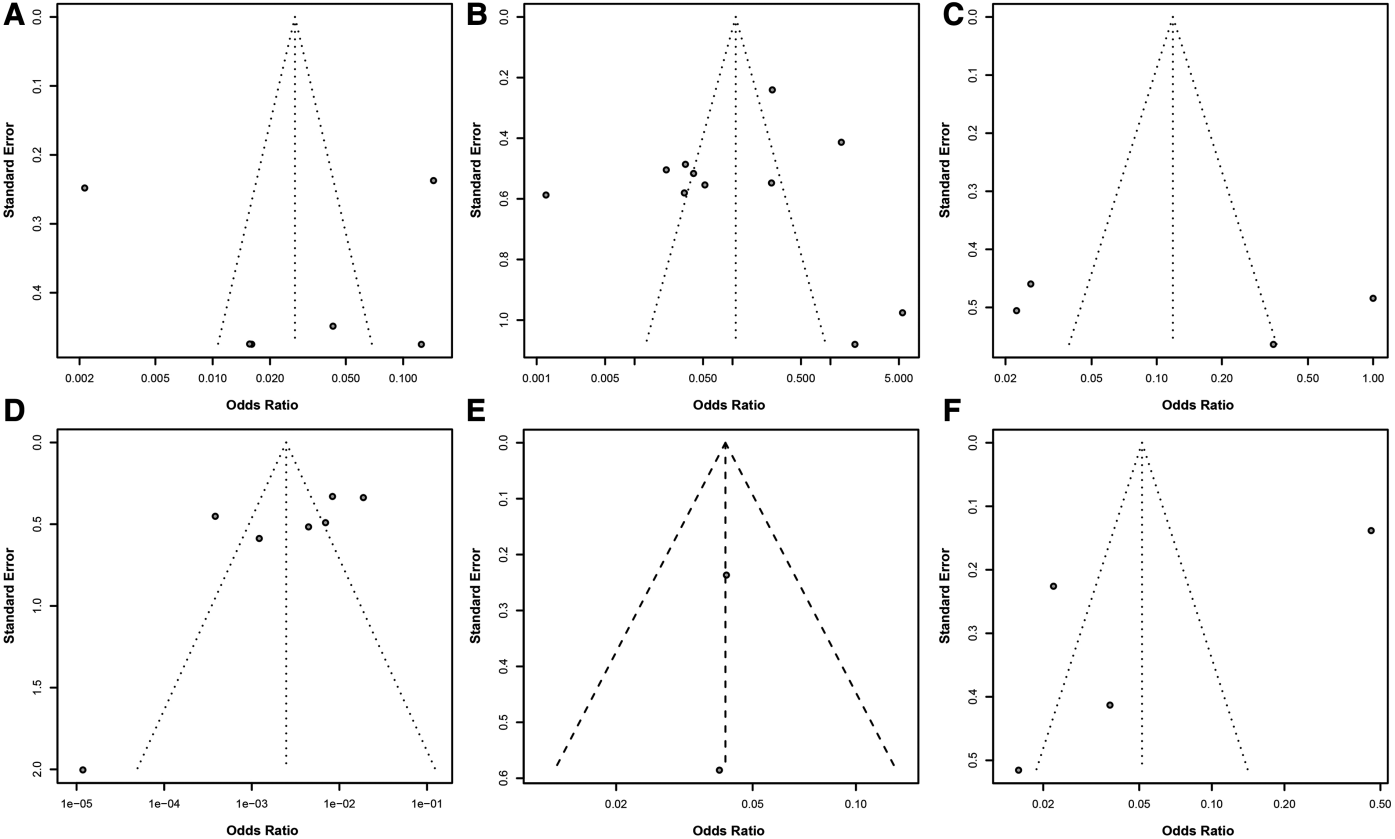
Assessment of publication bias in meta-analysis of risk factors for postoperative infection in colorectal cancer. **(A)** Funnel plot for BMI ≥24 kg/m^2^ as a risk factor. **(B)** Funnel plot for diabetes as a risk factor. **(C)** Funnel plot for preoperative low albumin levels as a risk factor. **(D)** Funnel plot for surgical methods as a risk factor. **(E)** Funnel plot for preoperative nutritional status as a risk factor. **(F)** Funnel plot for surgical duration exceeding 3 h as a risk factor. Each point in the funnel plot represents an estimate of the effect size and its precision for a study.

In a series of meta-analyses on risk factors for postoperative infection in colorectal cancer, funnel plots were utilized to assess publication bias (Figure 4). The analysis of these funnel plots revealed an excellent symmetry between the effect sizes and their standard errors for most studies, indicating a low risk of publication bias. While some studies deviated from the expected symmetric distribution, potentially reflecting heterogeneity among studies or the impact of specific study conditions, no evident one-sided skew or gaps were observed. This further supports the robustness and credibility of the meta-analysis results.

In summary, despite some heterogeneity among studies, the overall evidence suggests that the analysis linking these risk factors to the risk of postoperative infection in colorectal cancer is robust and highly credible. This provides critical guidance for clinicians in preoperative assessment and risk management, contributing to improved prevention and management of postoperative infections.

## Discussion

In recent years, changes in dietary patterns and lifestyle habits have led to colorectal cancer becoming a common malignancy within the gastrointestinal tract, with its incidence rate gradually increasing. Annually, approximately 1.2 million new cases and 600,000 deaths are attributed to this disease (50–52). Moreover, the age of onset has been trending younger (53). Colorectal cancer ranks fourth in incidence and second in mortality among all types of cancer worldwide, posing a serious threat to patient's health and safety (54–56). Currently, the preferred treatment for colorectal cancer patients is radical surgery to remove the lesion (57–59). Laparoscopic surgery, which allows for the visualization of surrounding tissues, nerves, blood vessels, and ureters, helps minimize damage to surrounding tissues and has shown significant clinical outcomes (60). However, patients with colorectal cancer are susceptible to the adverse effects of their condition, leading to poor physical health and reducing their capacity to undergo surgery (5, 38, 61). The high bacterial content and complex microbiota within the human colorectal cavity also increase the risk of postoperative incision infection (62, 63). During surgery, the spillage of intestinal contents can lead to the displacement and colonization of intestinal pathogens, resulting in a high rate of postoperative incision infections. Incision infections are common complications in clinical surgery and, in severe cases, can lead to systemic infections and sepsis, severely impacting postoperative recovery (64–66). Literature reports the rate of incision infections following colorectal surgery ranging from 2.7% to 26.0% (67–69). Colorectal cancer, being a debilitating disease, leads to a decline in patients' immune function, making them more prone to postoperative incision infections (70).

**Figure 5:**
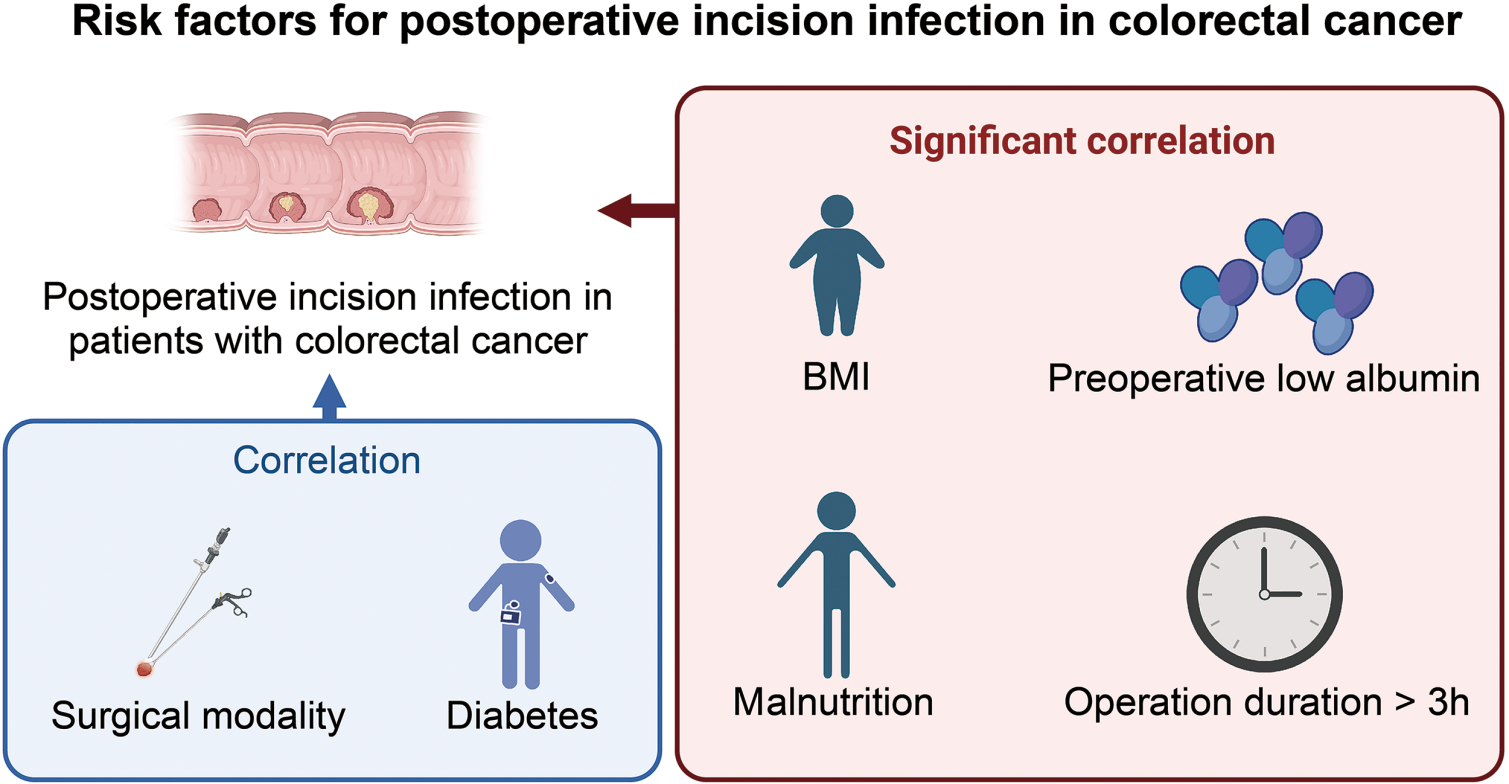
Illustration of risk factors for postoperative incision infection in colorectal cancer.

This study, through a systematic literature review and meta-analysis, delved into several potential risk factors for postoperative incision infection in colorectal cancer, including a BMI of ≥24 kg/m^2^, diabetes, preoperative low albumin levels, the method of laparoscopic surgery, preoperative malnutrition, and surgical duration exceeding 3 h. It identified that a BMI of ≥24 kg/m^2^, preoperative low albumin levels, preoperative malnutrition, and extended surgical duration are significant risk factors for postoperative incision infection, with diabetes also being a crucial risk factor. In contrast, laparoscopic surgery methods appear to be associated with a lower risk of infection (Figure 5). Our findings reveal a significant correlation between these factors and the risk of postoperative incision infection in colorectal cancer, offering essential insights for clinicians in preoperative assessment and postoperative management.

Colorectal cancer patients with a BMI greater than 24 kg/m^2^ are considered overweight. Overweight individuals have thick subcutaneous fat, which affects surgical visibility and is detrimental to surgical procedures. This can lead to increased surgical complexity, prolonged operation times, and subsequently raise the risk of postoperative wound infections. High fat content in overweight patients can inhibit the proliferation of immune cells, further increasing the risk of wound infections. Patients with a higher postoperative weight are more prone to abdominal fat liquefaction. The increased weight in patients is associated with hypertrophy of fat tissue and inadequate blood supply. Additionally, these patients commonly have chronic conditions such as hypertension and diabetes, which weaken their immune function and raise the probability of postoperative wound infections. The length of the incision is closely linked to the occurrence of postoperative infections. Overweight individuals have an increased likelihood of developing diabetes, altering their immune cell function and inflammatory response, thus making them more susceptible to surgical wound infections. Therefore, stringent perioperative infection prevention measures should be taken for patients with high BMI, and appropriate plans should be implemented before surgery to prevent postoperative infections. Previous studies have shown that a higher BMI in colorectal cancer patients increases the risk of surgical site infections. Hirao et al. found that when BMI is equal to or greater than 25 kg/m^2^, the incidence of incision infections significantly rises (OR = 2.28, 95% CI: 1.05–7.52). This finding is consistent with the results of this study. Research by Chen Yan and colleagues demonstrates that overweight patients experience compromised surgical visibility and operation due to the influence of subcutaneous fat. To achieve better visibility during surgery, incisions may need to be extended, increasing exposure and access. Postoperatively, incisions are more susceptible to liquefaction and necrosis, resulting in slower healing and an increased rate of surgical wound infections.

As societal lifestyles change and populations age, the incidence of diabetes is on the rise, leading to an increase in colorectal cancer patients with diabetes. These patients are more susceptible to postoperative incision infections due to immune system dysregulation and suppressed immune functions (40, 71, 72). Studies have shown that diabetes disrupts glucose metabolism, reduces glycolytic capacity, and weakens neutrophil migration, phagocytosis, and bactericidal functions (73). Protein synthesis decreases while degradation accelerates, reducing immunoglobulins' production, complements, and chemotactic factors, thereby diminishing immune function (74–76). The immune response in diabetic patients is relatively lower, and surgical trauma exacerbates glucose metabolism disorder. In a hyperglycemic environment, inflammation cell migration to the surgical site is hindered, further lowering the body's immunity and increasing infection risks, consistent with findings by Wukich et al. (77). The rate of incision infection in diabetic patients is significantly higher than in non-diabetic patients. The abnormal glucose metabolism in patients with diabetes impairs the normal function of inflammatory factors, facilitating pathogen colonization and growth in a high-glucose microenvironment, thus diminishing the patient's infection resistance.

Diabetic patients have microcirculation disorders, leading to a higher risk of anastomotic leakage post-surgery and potential abdominal infections. Diabetes-induced vascular plaque formation causes the narrowing of blood vessels, reducing tissue oxygenation, which can lead to tissue hypoxia, affecting oxidative-mediated microbial killing mechanisms and tissue oxygenation, and delaying tissue healing. Postoperative malnutrition is more likely in diabetic patients, adversely affecting recovery. Furthermore, wound healing in diabetic patients is slower. In healthy individuals, the metabolic level of glucose in diabetic patients is lower than usual, resulting in lower protein synthesis capacity and poorer cellular tissue repair abilities. Severe patients have impaired inflammatory cell function, affecting leukocyte phagocytosis. Immune function is below average, with fewer fibroblasts, hindering granulation tissue formation at the wound site, delaying wound healing, and even causing local edema. Surgical trauma can lead to postoperative stress-induced hyperglycemia, conducive to bacterial growth; a high glucose environment in the blood promotes bacterial colonization. Numerous studies have confirmed the impact of diabetes and perioperative hyperglycemia on surgical site infection. Hyperglycemia provides conditions for bacterial growth, and exudate in a high-glucose environment facilitates bacterial growth, reducing the body's immunity and leading to postoperative incision infections. Immune response functions are relatively lower in colorectal cancer patients with a history of diabetes. Post-laparoscopic surgery, surgical trauma further disrupts glucose metabolism, promoting inflammatory cell migration to the incision site, weakening immunity, and increasing the risk of postoperative incision infections. Persistent hyperglycemia in diabetic patients fosters bacterial growth, thereby increasing the rate of surgical site infections. Glucose metabolism disorder leads to a decreased pathogen clearance capacity, impaired immune function, and reduced infection resistance. Therefore, for colorectal cancer patients with diabetes, perioperative blood glucose management should be strengthened, aiming to keep blood glucose levels between 5.6–11.2 mmol/L, minimizing glucose fluctuations and thereby reducing the incidence of postoperative abdominal infections following colorectal cancer resection surgery.

Albumin levels directly reflect the nutritional status of the body (78–80). Low albumin levels indicate a higher risk of malnutrition, compromising immune function and increasing incision infection risk (81–83). Albumin, a significant component of human plasma proteins, is crucial in maintaining internal homeostasis (84). Low albumin levels reduce a patient's immunity, leading to drug absorption and metabolic disorders and complicating wound healing (85, 86). Therefore, clinical nutritional support should be intensified for such patients to boost their resistance, emphasizing the importance of preoperative nutritional interventions to enhance patient resilience (87, 88).

The most common complication following abdominal surgery is surgical site infections (SSIs), leading to increased postoperative pain, suffering, and economic burden, as patients require prolonged hospital stays, face readmissions, sepsis, and possibly death. This complication is associated with adverse economic consequences, increased morbidity, extended postoperative hospitalization, readmissions, sepsis, and mortality (89). Additionally, sepsis is a severe postoperative complication that can occur following colorectal surgery. It is typically associated with bacterial infections at the surgical site, which can lead to systemic inflammatory response syndrome (SIRS) and potentially progress to severe sepsis or septic shock if not promptly managed. Patients undergoing colorectal surgery are particularly vulnerable to sepsis due to the high bacterial load in the colon and potential intraoperative contamination. Early recognition and prompt management of sepsis are crucial for improving patient outcomes (89).

Surgical methods include traditional open surgery and laparoscopic surgery (90). Studies have shown that traditional open surgery, with its extensive trauma and significant blood loss, complicates postoperative recovery (91–93). Laparoscopic surgery, a significant advancement in modern science, has emerged as a new option for curative resection of colorectal cancer (67, 94, 95). It allows for precise observation of the surrounding tissue of the lesion, thus minimizing damage (96).

Open surgery requires an extended incision to ensure an excellent surgical field of view (97, 98). The larger incision, exposed to air for an extended period during surgery, significantly increases the risk of infection and may impact wound healing (99). Laparoscopic surgery facilitates precise observation of the lesion's surrounding tissues, nerves, blood vessels, and ureters, minimizing damage (100). With the advancement of laparoscopic techniques, pain post-colorectal cancer surgery has significantly reduced, and the recovery time has considerably shortened (101–103). The incision length in laparoscopic surgery is notably shorter than in open surgery, reducing skin integrity damage, bacterial displacement within the skin, and challenges in incision healing (104–107). Additionally, laparoscopic surgery, with its minimal tissue damage and smaller incisions, facilitates postoperative recovery, encouraging early patient mobilization to support wound healing and lower postoperative incision infection rates (105–107). Some studies have found that laparoscopic surgery minimally impacts human immune function and injury, making postoperative incision infections less likely (108, 109). Therefore, the choice of surgical method is particularly crucial, with a preference for laparoscopic surgery when possible (110, 111). Research indicates that in a single-center randomized controlled trial, the postoperative incision infection rate for patients undergoing laparoscopic surgery for colorectal cancer was 4.9% (47/961), significantly lower than the open surgery group (9.6%, 95/986) (112–115). This difference may be due to laparoscopic surgery reducing the direct contact between organs and environmental pathogens. Additionally, laparoscopic surgery avoids factors like peritonitis, increased intestinal permeability, and intestinal edema that are prone to surgical site infections, thereby lowering the rate of postoperative incision infections (116–118).

Nutritional status is a primary concern in the perioperative management of colorectal cancer patients (119, 120). Malnutrition lowers cellular and humoral immune responses, and correcting malnutrition can reduce the incidence of perioperative complications by up to 10% (121–123). The occurrence rate of perioperative complications is as high as 10% (124–126). Although no universal definition for diagnosing malnutrition, it typically encompasses conditions related to inadequate food intake, weight loss, and a low BMI (127–129). The European Society for Clinical Nutrition and Metabolism (ESPEN) defines malnutrition to include at least one of the following criteria: a weight loss of more than 10% of the original weight within six months, a BMI lower than 18.5 kg/m^2^, serum albumin less than 35 g/L, in the absence of liver or kidney dysfunction (130–135). Fujimichi et al. reported that malnutrition is an independent risk factor for postoperative incision infection in colorectal cancer patients (OR = 2.52, 95%, *p* = 0.01) (42, 136, 137). Furthermore, a registry study at the Hokeland University Hospital in Norway showed that among 1,194 patients undergoing surgical treatment, those at nutritional risk were more likely to develop incision infections, with a positive correlation between the incidence of incision infections and nutritional risk (OR = 1.81, *p* = 0.047) (138–141). ESPEN recommends that severely malnourished patients scheduled for major gastrointestinal surgery should receive preoperative nutritional support for 10–14 days. Enteral nutrition should be the first choice if there are no contraindications (142–145). Enhancing perioperative nutrition and supportive care is crucial for malnourished patients, ensuring sufficient energy and nutrient intake to prevent perioperative incision infections. Malnourished colorectal cancer patients often have electrolyte imbalances, anemia, and lower immunity, increasing the risk of postoperative incision infections. Patients should receive enteral nutrition as soon as gastrointestinal recovery permits, maintaining the intestinal barrier and immune barrier, reducing endotoxin absorption and intestinal flora displacement, thereby providing a conducive internal environment for wound healing (49, 146–150).

This study's findings indicate that a surgical duration exceeding three hours is a risk factor for surgical site infections in patients with colorectal cancer, aligning with Katsuno's research. It has been shown that the risk of postoperative incision infection in colorectal cancer increases with the length of the surgery (151–154). Extended surgical times are often associated with increased blood loss, potentially leading to tissue hypoxia (155–157). Longer surgeries inevitably carry a higher risk of bleeding and increased blood loss, reducing the body's resistance and inducing infection (158). The longer the surgery, the more energy the patient expends, raising the risk of exogenous infection and, thereby, the risk of postoperative incision infection (159–161). Prolonged surgical duration also means the sterile environment within the abdomen is exposed to air for a longer time (162–164). Even in an operation meeting standard requirements, air cleanliness decreases with extended surgical times, increasing the probability of local bacterial contamination (165–167). The wound's exposure to air also increases, leading to a higher bacterial count at the incision site and increasing the possibility of tissue cell destruction. Longer surgeries, extended exposure to tissue traction, and prolonged use of surgical energy devices can damage tissues. Moreover, extended anesthesia can adversely affect the patient's immune function. The body's immunity diminishes as anesthesia duration and intraoperative blood loss increase. The length of the surgery is not only related to the patient's physical condition but also largely depends on the surgeon's skill and proficiency in the operation. Thus, an increased rate of postoperative incision infection indicates that more complex, challenging, and traumatic surgeries with longer durations lead to higher infection rates (168, 169). Therefore, enhancing the surgical skills and intraoperative proficiency of surgeons, reducing surgical trauma, shortening surgical duration, and lowering the incidence of incision infections is paramount. Zheng Hui's multivariate analysis of 2,308 patients showed that surgical duration (OR = 1.007, 95% CI: 1.002–1.012) is an independent risk factor for incision infection (170). Thus, effectively controlling surgical duration can significantly reduce the incidence of incision infections (171–173).

In addition to risk assessment, advancements in deep learning algorithms hold significant potential for improving the accuracy and efficiency of colorectal cancer (CRC) diagnosis. Deep learning models can analyze histopathology images with high precision, aiding in the classification and diagnosis of CRC. These algorithms can learn complex patterns from large datasets, potentially outperforming traditional diagnostic methods. Further investigation into the clinical implementation of these algorithms could enhance CRC detection's accuracy and efficacy (174, 175).

Based on the analysis of various risk factors, future clinical practices can implement the following strategies to prevent postoperative incision infections in patients with colorectal cancer:

(1) Preoperative: Implement infection prevention measures and avoid scheduling surgeries during summer. For diabetic patients, intensify monitoring and control of blood glucose levels and use insulin judiciously. Surgery should proceed only when blood glucose levels are within normal ranges. For elderly patients, complications should be vigilantly monitored and actively managed preoperatively. Additionally, patients' nutritional status should be assessed, and timely nutritional support should be provided to those with low serum albumin to ensure balanced daily nutrient intake and optimal preoperative nutrition.
(2) Intraoperative: Adhere to standard sterile procedures, minimize electrosurgical use in patients with thick adipose layers, and adjust the electrosurgical power as necessary. Inactive fatty tissue should be rinsed with saline during incision closure. Moreover, surgical preparations should be meticulously planned, requiring close cooperation among medical staff to enhance procedural proficiency and actively manage surgical duration. When appropriate, consider laparoscopic surgery for its reduced patient trauma and lower postoperative incision infection rates, taking into account the patient's specific health status and condition.
(3) Postoperative: Monitor changes in patient vitals, replenish energy promptly as needed, and encourage high-fiber and protein-rich foods to boost nutrition and maintain electrolyte balance. Pay attention to changes in the nature, volume, and color of drainage fluid, replace drainage bags timely to prevent incision-related infections, and regularly change wound dressings to prevent bacterial growth and infection.

Despite our study's rigorous design and execution, it has limitations. First, the significant heterogeneity among studies may affect the robustness of our conclusions, although sensitivity analysis and publication bias assessment have been conducted to ensure the reliability of the results. Second, the quality of included studies varies, and despite rigorous evaluation using the Cochrane risk of bias tool and NOS scoring system, the impact of low-quality studies must be partially ruled out. Additionally, language and database search limitations might have led to selection bias due to potentially relevant studies needing to be included.

Future research should further explore the causal relationships between these risk factors and postoperative incision infections in colorectal cancer and how they interact with other potential risk factors. Moreover, as medical technology advances, new surgical techniques and postoperative management strategies may influence the risk of postoperative incision infections in colorectal cancer. Therefore, ongoing research and updated meta-analyses must ensure our conclusions reflect the latest scientific evidence.

In summary, this study provides critical insights into the risk factors for postoperative incision infection in colorectal cancer, emphasizing the importance of comprehensive assessment and management of these risk factors in clinical practice. By early identification and intervention of these risk factors, the incidence of postoperative incision infections in colorectal cancer patients can potentially be reduced, thereby improving patient prognosis and quality of life. Future research should aim to explore additional potential risk factors and evaluate the effectiveness of various prevention and management strategies to optimize postoperative care for colorectal cancer patients further.

## Data Availability

The original contributions presented in the study are included in the article/Supplementary Material, further inquiries can be directed to the corresponding author.
